# The femoral intercondylar notch is an accurate landmark for the resection depth of the distal femur in total knee arthroplasty

**DOI:** 10.1186/s43019-022-00159-x

**Published:** 2022-07-07

**Authors:** David W. Liu, Sara Martinez Martos, Yifei Dai, Elaine M. Beller

**Affiliations:** 1 Gold Coast Centre for Bone and Joint Surgery, 14 Sixth Avenue, Palm Beach, QLD 4221 Australia; 2 Exactech Inc., 2320 NW 66th Ct., Gainesville, FL 32653 USA; 3 Centre for Research in Evidence-Based Practice, Bond University, 14 University Drive, Robina, QLD 4226 Australia

**Keywords:** Total knee arthroplasty, Distal femoral resection, Intercondylar notch

## Abstract

**Introduction:**

Conventionally, the depth of distal femoral resection in total knee arthroplasty is referenced from the most prominent distal femoral condyle. This surgical technique does not consider pathological alterations of articular surfaces or severity of knee deformity. It has been hypothesized that the femoral intercondylar notch is a clinically reliable and more accurate alternative landmark for the resection depth of the distal femur in primary total knee arthroplasty.

**Methods:**

The resection depths of the distal femur at the medial and lateral femoral condyles and intercondylar notch were measured using computer navigation in 406 total knee arthroplasties. Variability between the bone resection depths was analyzed by standard deviation, 95% confidence interval and variance. Clinical follow-up of outcome to a minimum of 12 months was performed to further inform and validate the analysis.

**Results:**

Mean resection depth of the medial condyle was 10.7 mm, of the lateral condyle 7.9 mm and of the femoral intercondylar notch 1.9 mm. The femoral intercondylar notch had the lowest variance in resection depth among the three landmarks assessed, with a variance of 1.7 mm^2^ compared to 2.8 mm^2^ for the medial femoral condyle and 5.1 mm^2^ for the lateral femoral condyle. The intercondylar notch reference had the lowest standard deviation and 95% confidence interval. The resection depth referencing the notch was not sensitive to the degree of flexion contracture pre-operatively, whereas the medial and lateral condyles were. For varus deformed knees, distal femoral resection depth at the notch averaged 2 mm, which corresponds to the femoral prosthesis thickness at the intercondylar region, while for valgus deformed knees, the resection was flush with the intercondylar notch.

**Conclusions:**

The femoral intercondylar notch is a clinically practical and reproducible landmark for appropriate and accurate resection depth of the distal femur in primary total knee arthroplasty.

**Level of evidence:**

Level III: Retrospective cohort study.

## Introduction

The distal femoral resection in primary total knee arthroplasty (TKA) has a direct impact on the extension gap and femoral joint line, which in turn influences range of motion, collateral stability and function following TKA [1, 3, 4, 12]. Under-resection leads to residual fixed flexion deformity, resulting in quadriceps’ mechanical disadvantage, altered tibio-femoral and patello-femoral force distribution and inferior patient-reported outcome [17]. On the other hand, over-resection may culminate in mid-flexion instability and patella height problems [3, 7]. Traditionally, the femoral condyles are used to reference the magnitude of distal resection depth, aiming for the same amount of bone as prosthesis thickness from the most prominent condyle. However, inaccuracies may occur with this method depending on the amount of condylar wear and degree of coronal deformity or flexion contracture [9, 13, 14].

It has been the senior author’s observation when using computer navigation for TKA that the thickness of the registered distal femoral osteotomy is more consistent at the intercondylar notch region. We consequently performed this study to investigate whether the femoral intercondylar notch is a more constant indicator of appropriate distal femoral resection magnitude than the customary prominent medial or lateral condyle. The femoral intercondylar notch has potential advantages over condylar referencing for the resection depth: (1) it is a midline point not affected by the degree of knee deformity or distal femoral valgus angle; and (2) the intercondylar region normally displays native thickness of articular cartilage even in the presence of significant condylar wear [2, 6]. An additional advanage is that the intercondylar notch is a clearly visible, accessible and reproducible landmark often used to help determine femoral component rotation [5, 11].

Previous studies have reported that the femoral sulcus can be used to determine the desirable level of distal femoral resection in TKA, but both were radiological studies using simulation or planning software only [10, 18]. In the study reported here, we examined registered bone resection depths intra-operatively using computer navigation following TKA preparation, with clinical follow-up of patient outcome also recorded.

The first aim of this study was to analyze the resection thickness of the medial and lateral condyles and intercondylar region of the distal femur using computer navigation in a group of patients with well-functioning TKAs with a minimum 1-year follow-up. The second aim was to determine if the intercondylar notch could be used accurately to gauge distal resection in primary TKA. Our study hypothesis is the femoral intercondylar notch is a clinically reliable landmark for the resection depth of the distal femur in primary TKA.

## Methods

We undertook a retrospective study of 406 patients who received primary TKA from April 2014 to January 2016 using computer navigation and had been followed up for a minimum of 1 year. Prospective data were collected on patient-reported outcome, satisfaction and range of motion of the knee at a minimum of 1 year following surgery. Institutional review board approval was obtained for the study (Protocol Approval No. 13/24), and all participating subjects provided a signed consent form after they had read the study patient information sheet and the nature of the study had been fully explained. All patients who underwent primary TKA during the study period were recruited for inclusion. Exclusion criteria included patients with previous surgery except for arthroscopy on the knee or previous fracture to the study limb and patients who subsequently underwent revision surgery following their primary TKA. The inclusion/exclusion criteria were aimed to only include satisfied patients with well-functioning TKAs and exclude patients with significant intra-articular or extra-articular deformities.

**Fig. 1:**
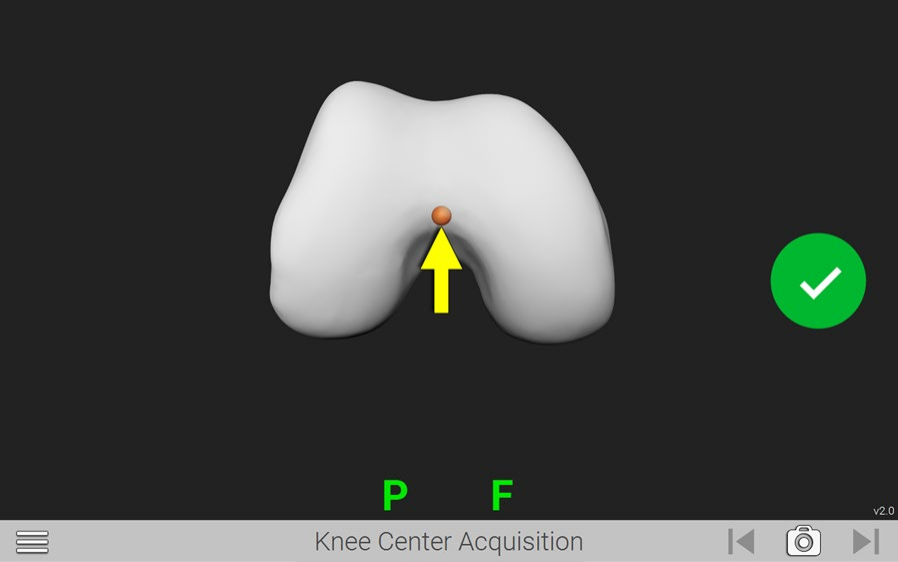
Femoral intercondylar notch landmark. Articular surface landmark for measurement of the resection depth at the femoral intercondylar notch corresponds to the point for knee centre acquisition required for registration during knee navigation

**Fig. 2:**
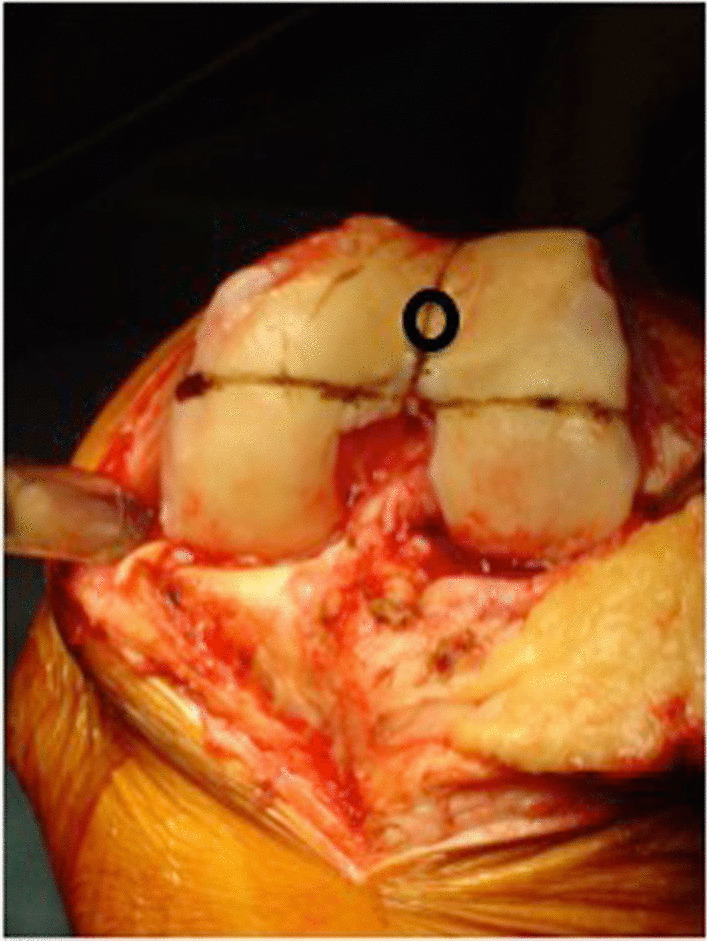
Clinical landmark for femoral intercondylar notch. The point representing the knee center, from which the notch resection depth was calculated, is equivalent to the entry point for an intramedullary guide rod. It is a routinely identified landmark, familiar to all surgeons performing total knee arthroplasty

One surgeon (senior author), using a combination of regional and/or general anesthesia, performed all surgeries using computer navigation. The knee was exposed through a medial parapatellar approach without tourniquet application. Anatomical landmarks were recorded using the knee navigation system. The intra-operative surgical landmark chosen for the femoral intercondylar notch reference corresponded to the knee centre of the distal femur routinely captured in the navigation workflow (Fig. 1). In the senior author’s surgical technique, the knee center point is located in the deepest point of the trochlea sulcus of the distal femur, typically slightly medial to the midpoint of the condyles (Fig. 2).

All knees were operated on using a modified gap balancing technique aiming for neutral mechanical alignment. Measured resection of the distal femur and proximal tibia was performed, followed by gap balancing of the flexion gap using a calibrated tensioner device. During femoral component sizing, flexion gap recreation was prioritized. The calibrated flexion space tensioner device incorporated a femoral sizing gauge using a navigation tracker, which allowed real-time feedback on femoral component position relative to both the anterior and posterior references. The femoral component size and antero-posterior (AP) position could therefore be adjusted with real-time feedback on the navigation unit of component location relative to both anterior and posterior references and flexion gap size. Priority was given to restoring and equalizing the flexion gap to extension gap through a combination of femoral component size and AP alignment adjustments.

The distal femoral resection was referenced from the most prominent distal femoral condyle, aiming for equivalent thickness as the prosthesis. In the majority of cases, the medial condyle was most prominent, even in varus aligned knees due to the distal femoral valgus angle. Occasionally with significant medial femoral condylar wear and a low distal femoral valgus angle, the lateral condyle was most prominent and therefore used to gauge the resection. The proximal tibial resection referenced the highest point of the most prominent condyle, aiming for equivalent thickness as the thinnest polyethylene insert. A cruciate-retaining trial prosthesis was inserted, ensuring full passive extension and ligament balance. If full extension with the trial components in place was not achieved after posterior osteophyte clearance and posterior capsular release, additional distal femoral bone resection was performed until full extension was achieved. The final plane of the distal femoral cut was registered and recorded using the navigation system after removal of the accepted trial implants.

**Fig. 3:**
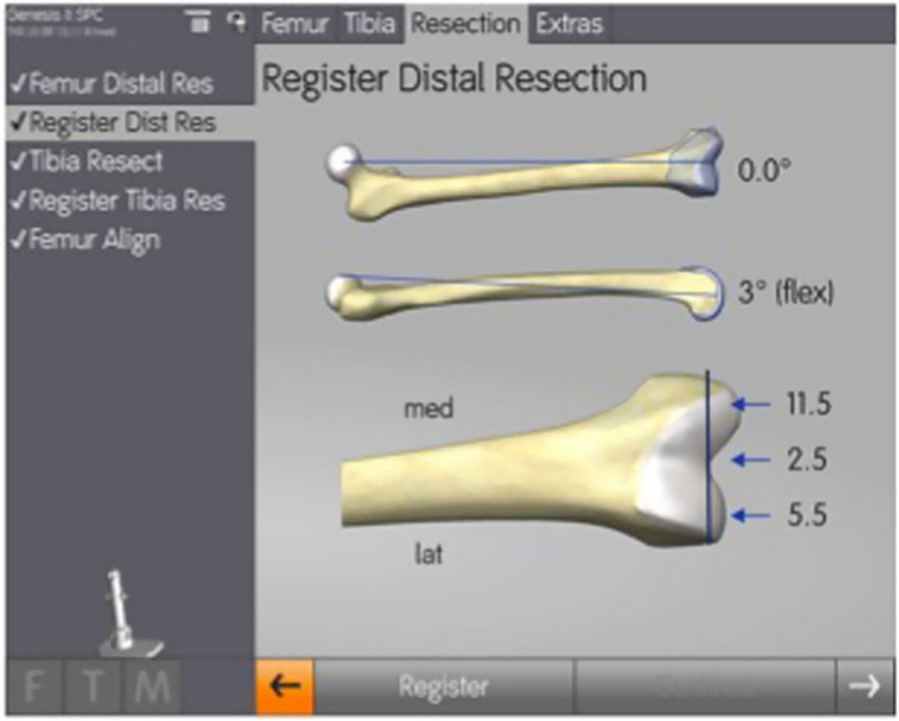
Distal resection depth registration. Digitization of the final distal femoral resection after satisfactory balance and range of motion was achieved and removal of the trial components. The resection depths at the three regions of interest are depicted

The final registered resection depths referencing the medial and lateral femoral condyles, and the intercondylar region of the distal femur, as well as the varus valgus angulation of the distal femoral resection, were extracted from the knee navigation surgical log files. The measurement screen on the navigation system used to record resection depths for each of the three regions is shown in Fig. 3. For the intercondylar notch measurement, the resection thickness corresponded to the distance between the knee center landmark and the digitized resection plane. For the medial and lateral condyles, the navigation software recorded the distance from the most distal point on each condyle to the recorded resection plane.

Patient demographic data were obtained from the medical charts. All patients completed the Oxford Knee Score (OKS) and Knee Satisfaction Score questionnaires at a minimum of 12 months post-operatively. Range of motion of the operative knee was analyzed at 12 months of follow-up using a handheld goniometer.

Descriptive statistical analysis of the data was performed and presented as counts and percentages for categorical variables and means, ranges and standard deviations for continuous variables. Variability between the distal resection bony landmarks was compared using standard deviations, 95% confidence interval and variance.

Correlation between a quantitative variable with an ordinal variable were assessed using Spearman’s rho analysis. A *P* value < 0.05 was considered to be statistically significant.

## Results

**Fig. 4:**
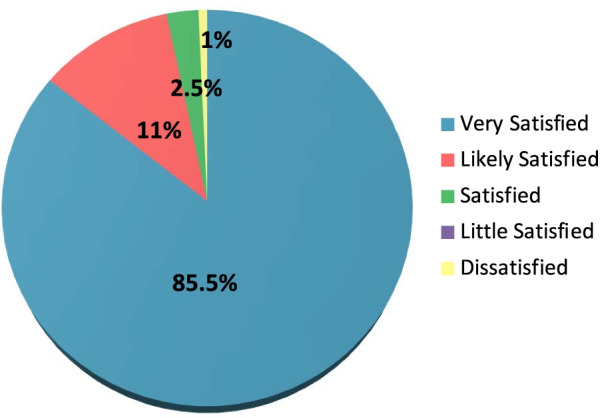
Patient satisfaction at the 12-month follow-up. Of the 406 patients in the study population, 86% patients were completely satisfied and only 1% were dissatisfied at the 12-month follow-up

Demographics of the study population are summarized in Table 1. The majority of patients achieved an acceptable result at 12 months post-operatively, with 85.5% completely satisfied, but equally important only 1% were dissatisfied (Fig. 4). Most patients achieved full extension at 12 months, with 3.2% of patients displaying residual fixed flexion of ≥ 5 ° and no patient having > 10 ° flexion contracture at 12 months. The 12-month outcome data are outlined in Table 2.

**Table 1:** Patient demographics and pre-operative details of the study population

Patient demographics and pre-operative details	Values
Number of patients	406
Age (years)	70.2 ± 9.4 Range 45–93
Gender (%)	51.5% female 48.5% male
Body mass index (kg/m^2^)	29.0 ± 4.7 Range 18.1–47.1
Side	51.9% Right 48.1% Left
Pre-operative deformity (%)	77.4% Varus 22.6% Valgus
Pre-operative mechanical alignment (°)	3 varus ± 5.4 Range 12 varus to 17 valgus
Pre-operative extension (°)	5.6 ± 6.3 Range -23–30
Pre-operative flexion (°)	125 ± 10.6 Range 80–149

Data are presented as the mean ± standard deviation (SD), with the range, unless indicated otherwise

**Table 2:** Post-operative outcome measures of patient group at the 12-month follow-up, expressed as mean with standard deviation and range

Post-operative outcome measures	
Oxford knee score at the 12 month follow-up	42.8 ± 4.32 Range 25–48
Post-operative coronal mechanical alignment (°)	Varus 0.69 ± 1.39 Range − 7–6
Post-operative extension (°)	0.28 ± 3.55 Range 0–10
Post-operative flexion (°)	116.5 ± 7.35 Range 85–130
Patients with residual flexion contracture ≥ 5 ° (%)	3.1%

Data are presented as the mean ± SD, with the range, unless indicated otherwise

The registered resection thickness of the medial condyle, the lateral condyle and the intercondylar notch of the distal femur are detailed in Table 3. Of the study cohort, 66 patients (16.3%) required an additional distal femoral recut after initially referencing the most prominent condyle, to provide an adequate extension gap and correct fixed flexion with the trial components in situ. The femoral intercondylar notch demonstrated the least variability of the three anatomical references for final resection depth, with the lowest standard deviation, 95% confidence interval and variance. Additionally, the mean eventual thickness of the medial condyle was greater than the 9-mm prosthesis thickness while the mean thickness of the lateral condyle was less than 9 mm. The resection depth of the distal femur at the intercondylar region was not affected by the degree of pre-operative fixed flexion (*P* = 0.302), unlike the medial femoral condyle (*P* = 0.004) and lateral femoral condyle (*P* = 0.023) which were affected (Table 4).

**Table 3:** Final resection depths of the distal femur for each region of interest (absolute distance relevant to the actual bony resection)

Region	Depth		
	Mean ± SD (mm)	95% CI (mm)	Variance (mm^2^)
Medial femoral condyle	10.7 ± 1.7	[7.3, 14.0]	2.8
Lateral femoral condyle	7.9 ± 2.3	[3.3, 12.4]	5.1
Femoral intercondylar notch	1.9 ± 1.3	[0.0, 4.5]	1.7

CI Confidence interval

**Table 4:** *P*-values for correlation between resection thickness at each region of interest and patient, clinical and prosthetic variables

Variable	Medial femoral condyle	Lateral femoral condyle	Femoral intercondylar notch
Age	0.08	0.15	0.07
Gender	0.003^*^	0.03*	0.09
Body mass index	0.35	0.12	0.34
Deformity	0.46	0.06	0.01*
*Fixed flexion*			
< 5 °	0.143	0.023*	0.214
> 5 °	0.004^*^	0.454	0.302
Femoral size	0.90	0.93	0.17

*Statistically significant difference at *P-*value < 0.05

However, the intercondylar notch resection depth was associated with the type of pre-operative coronal deformity of the knee. Sub-analysis revealed that in varus knees, resection depth at the intercondylar region averaged 2 mm, which is equivalent to the 2 mm thickness of the metal of the prosthesis in the same region. In valgus knees, the femoral intercondylar resection thickness was 0.5 mm or essentially just skimming the cartilage of the sulcus. The difference in the intercondylar notch resection depth between varus and valgus knees was statistically significant (*P* = 0.01).

## Discussion

The most important finding of the present study is that the femoral intercondylar notch is a more accurate and reproducible landmark than the traditional medial or lateral condyle in guiding resection depth of the distal femur in primary TKA. The femoral intercondylar notch had the lowest variability of the three landmarks used, which appears to be clinically relevant. The resection depth at the notch was not sensitive to degree of flexion contracture pre-operatively; it was, however, significantly different for varus as opposed to valgus knees. We observed that in varus knees the bone resection depth at the notch corresponded to the thickness of the femoral implant at the intercondylar region, while in valgus knees the resection was level with the intercondylar notch. Importantly, the mean thickness of medial condylar resection was 10.7 mm, which is greater than the 9-mm thickness of the prosthesis. Therefore, had the condyles been used to guide distal femoral resection, our data suggest that under-resection, potentially leading to a tight extension gap, would have occurred. Using the intercondylar notch as reference may potentially avoid the inconvenience and difficulties of re-cutting the distal femur with measured resection technique if the extension gap is tight, or the disadvantages of significant proximal shift of the femoral joint line if using the gap-balancing technique.

Previous authors have reported on using the intercondylar region for the distal femoral osteotomy. Using computed tomography(CT)-based navigation planning software, Kuriyama et al. found in 40 varus knees that the deepest subchondral bone of the trochlea groove was a predictable distance from the medial and lateral distal condyles [10]. These authors recommended the sulcus cut technique be used to determine the desirable level of distal femoral resection in TKA. Yue et al. examined the mean distance from the distal surface of the femoral condyles to the intercondylar notch ceiling in 100 healthy Chinese subjects using three-dimensional models constructed using CT scans [18]. The mean distance was consistent and did not differ with varying sizes of the distal femur. These authors advocated that the distal femoral bone cut should be at the level of the intercondylar notch ceiling. Both studies were based on radiological data, which is a surrogate measurement of clinical practice and may be sensitive to image quality and inter-observer variability. Our study adds to the literature on this concept by providing intra-operative and clinical information and validation in a large cohort of patients with 12-month follow-up. The findings support clinical use of the intercondylar notch as a reliable landmark for the distal femoral resection depth in primary TKA. The present study contributes further information to assist surgeons refine the sulcus or intercondylar notch technique, such as the effect of varus-valgus deformity in varying the resection depth.

Our study has several weaknesses and limitations. First, our patient cohort consisted of Caucasian patients residing in Australia and may not be applicable across all ethnicities or other regions of the world. Second, we did not consider the presence of trochlea dysplasia nor measure the effect of sulcus angle on the results in our patient cohort. However, trochlea dysplasia usually affects the proximal part of the femoral trochlea and the landmark described in our study is located in the distal part of the intercondylar sulcus, which is often normal even with trochlea dysplasia [16]. Being a midline point in the distal groove, the sulcus angle is unlikely to have an impact on the resection depth in this region. Third, as one surgeon performed all cases, various surgical techniques and preferences were not investigated. The target in all cases was mechanical alignment, and the findings may not be appropriate with alternative alignment techniques, such as kinematic alignment. During the present study, in the cases where there was residual flexion contracture with the trial components after posterior recess clearance, additional distal femoral bone was resected to increase the extension gap, whereas the error may have been due to under-resection of the proximal tibia. However, most of our patients reported satisfactory outcomes at 12 months and there were no revisions for instability in the cohort. We did not have sufficient statistical power to determine at the 12-month follow-up whether the notch resection depth was different between satisfied and dissatisfied patients, and between those with full extension compared with residual fixed flexion. The number of dissatisfied patients was too low and only 3.2% of patients did not achieve full flexion. Another limitation to the study is that computer navigation was used in all cases; surgeons not using navigation may feel the findings are not applicable to mechanical instrumentation. We argue however that the intercondylar landmark is easily identified and familiar to all surgeons and that the principle can be transferred to all surgical techniques. The notch reference can be adapted to mechanical instrumentation by using an angle wing through the distal cut block or potentially redesigning mechanical instruments to measure from this landmark. It is also easily adaptable to patient-specific instrument techniques, as shown in the studies of Kuriyama [10] and Yue [18].

We are not advocating that the femoral notch reference is 100% accurate and foolproof. While the intercondylar notch had the lowest variance, surgeons should still also consider resection thickness of the condyles in conjunction with the notch in deciding the distal femoral cut. Nevertheless, the authors believe that this study presents important findings and may assist surgeons in deciding the optimum resection depth of the distal femur in primary TKA. The authors advocate for the use of the femoral intercondylar notch as an additional reference landmark that is easily and readily accessible intra-operatively.

## Conclusion

The femoral intercondylar notch is an accurate, reproducible, and clinically practical landmark for the distal femoral resection depth in primary total knee arthroplasty.

## Data Availability

The datasets used and/or analysed during the current study are available from the corresponding author on reasonable request.

## Abbreviations

CT: Computed tomography
OKS: Oxford Knee Score
TKA: Total knee athroplasty
